# A Paravermal Trans-Cerebellar Approach to the Posterior Fossa Tumor Causes Hypertrophic Olivary Degeneration by Dentate Nucleus Injury

**DOI:** 10.3390/cancers13020258

**Published:** 2021-01-12

**Authors:** Martin A. Schaller-Paule, Peter Baumgarten, Volker Seifert, Marlies Wagner, Eike Steidl, Elke Hattingen, Felix Wicke, Joachim P. Steinbach, Christian Foerch, Juergen Konczalla

**Affiliations:** 1 Department of Neurology, University Hospital Frankfurt, Goethe-University, 60528 Frankfurt am Main, Germany; foerch@em.uni-frankfurt.de; 2 Department of Neurosurgery, University Hospital Frankfurt, Goethe-University, 60528 Frankfurt am Main, Germany; peter.baumgarten2@kgu.de (P.B.); volker.seifert@kgu.de (V.S.); juergen.konczalla@kgu.de (J.K.); 3 Institute of Neuroradiology, University Hospital Frankfurt, Goethe-University, 60528 Frankfurt am Main, Germany; marlieswa@gmx.de (M.W.); eike.steidl@kgu.de (E.S.); elke.hattingen@kgu.de (E.H.); 4 University Cancer Center Frankfurt (UCT), University Hospital Frankfurt, Goethe-University, 60528 Frankfurt am Main, Germany; joachim.steinbach@kgu.de; 5 German Cancer Consortium (DKTK), Partner Site Frankfurt/Mainz, 60528 Frankfurt am Main, Germany; 6 Department of Psychosomatic Medicine and Psychotherapy, Johannes Gutenberg University Mainz, 55131 Mainz, Germany; wicke@allgemeinmedizin.uni-frankfurt.de; 7 Dr. Senckenberg Institute of Neurooncology, University Hospital Frankfurt, Goethe-University, 60528 Frankfurt am Main, Germany; 8 Frankfurt Cancer Institute (FCI), University Hospital Frankfurt, Goethe-University, 60528 Frankfurt am Main, Germany

**Keywords:** cerebellum, cerebellar mutism, neurosurgery, HOD, CMS, medulloblastoma

## Abstract

**Simple Summary:**

Posterior fossa tumor surgery is challenging due to the proximity and exposure of cerebellar structures. A favorable operative approach is unknown. Following lesions to the dentato–rubro–olivary-pathway, a neurodegenerative disease called hypertrophic olivary degeneration (HOD) can occur. This study for the first time demonstrates that paravermal trans-cerebellar approaches are associated with a significantly higher likelihood of HOD on MRI when compared to other approaches. This finding can well be attributed to dentate nucleus (DN) injury. Furthermore, cerebellar mutism syndrome (CMS) was discussed in the literature to be correlated with HOD due to a functional overlap of pathways involved. We found no such correlation in this study, but HOD was shown to be a reliable indicator for surgical disruption of efferent cerebellar pathways involving the DN. Henceforth, neurosurgeons should consider more midline or lateral approaches in posterior fossa surgery to spare the DN whenever feasible, and focus on cerebellar functional anatomy in their preoperative planning.

**Abstract:**

*Background:* In brain tumor surgery, injury to cerebellar connectivity pathways can induce a neurodegenerative disease called hypertrophic olivary degeneration (HOD), along with a disabling clinical syndrome. In children, cerebellar mutism syndrome (CMS) is another consequence of damage to cerebello–thalamo–cortical networks. The goal of this study was to compare paravermal trans-cerebellar to other more midline or lateral operative approaches in their risk of causing HOD on MR-imaging and CMS. *Methods:* We scanned our neurosurgical database for patients with surgical removal of pilocytic astrocytoma, ependymoma and medulloblastoma in the posterior fossa. Fifty patients with a mean age of 22.7 (±16.9) years were identified and analyzed. *Results:* HOD occurred in *n* = 10/50 (20%) patients within four months (median), always associated with contralateral dentate nucleus (DN)-lesions (*p* < 0.001). Patients with paravermal trans-cerebellar approach significantly more often developed HOD (7/11; 63.6%) when compared to other approaches (3/39; 7.7%; *p* < 0.001). Injury to the DN occurred more frequently after a paravermal approach (8/11 vs. 13/39 patients; *p* < 0.05). CMS was described for *n* = 12/50 patients (24%). Data indicated no correlation of radiological HOD and CMS development. *Conclusions:* A paravermal trans-cerebellar approach more likely causes HOD due to DN-injury when compared to more midline or lateral approaches. HOD is a radiological indicator for surgical disruption of cerebellar pathways involving the DN. Neurosurgeons should consider trajectories and approaches in the planning of posterior fossa surgery that spare the DN, whenever feasible.

## 1. Introduction

In posterior fossa tumor surgery, a lesion in the cerebellar dentate nucleus (DN) can trigger a series of trans-synaptic neurodegenerative events that ultimately lead to a hypertrophic olivary degeneration (HOD) of the inferior olivary nucleus in the medulla [1,2,3,4]. Previous clinical case series have indicated that surgical injury to the DN may be more closely associated with HOD occurrence than an invasive tumor growth [5,6]. However, which specific operative approaches constituted a risk factor remained unclear.

**Figure 1:**
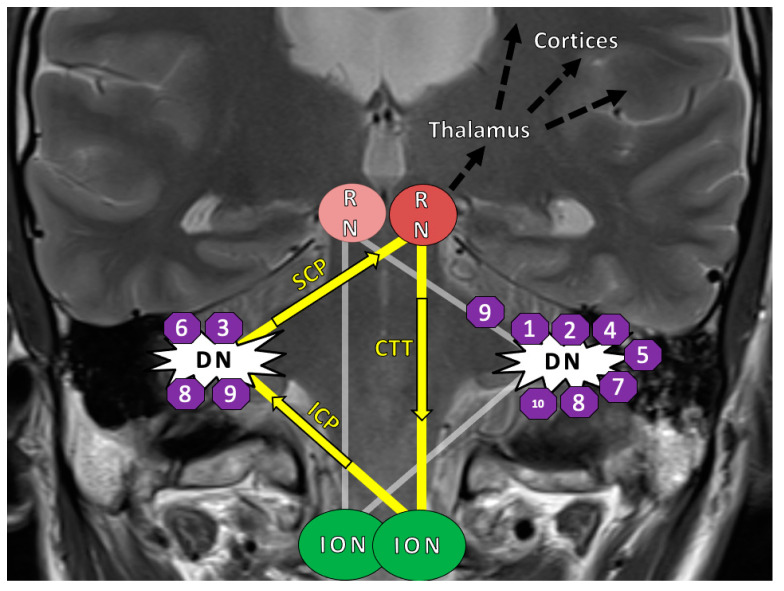
Disease model with lesion sites: The Guillain–Mollaret-triangle (yellow) and its unidirectional course of inhibitory fibers that ascent from the dentate nucleus (DN) to the contralateral red nucleus (RN) and then descend ipsilateral to the inferior olivary nucleus (ION). The bilateral overlapping Guillain–Mollaret triangles (yellow and grey) together form a “tilted star of David configuration” [5]. The numbers in octagons indicate the position of the operative lesions in Patients 1–10 of this study, who ultimately developed a HOD appearance on MRI. In bilateral lesions, octagons are shown on both sides. Fibers of the GMT, which is also called the dentato–rubro–olivary-pathway, overlap with dentato–thalamo–cortical pathways proceeding into both hemispheres (black arrows) [10]. Abbreviations: GMT = Guillain–Mollaret-triangle; HOD = hypertrophic olivary degeneration; SCP = superior cerebellar peduncle; ICP = inferior cerebellar peduncle; RN = red nucleus; CTT = central tegmental tract; DN = dentate nucleus; ION = inferior olivary nucleus.

The HOD occurs after lesions to a functional loop called the Guillain–Mollaret triangle (GMT), which is composed of three anatomic structures: the red nucleus (RN) in the mesencephalon, the ipsilateral inferior olivary nucleus (ION) in the medulla and the contralateral dentate nucleus (DN) in the cerebellum (Figure 1) [5,7,8,9]. It is also referred to as the dentato–rubro–olivary pathway (DROP).

The HOD itself is a secondary neurodegenerative disease: over the course of months, it leads to a characteristic clinical syndrome of rhythmic palatal tremor, (dentato–rubral) Holmes-tremor and vertical pendular nystagmus [11,12,13]. Patients may be severely impaired by the progressing neurological symptoms and are at risk of developing dysphagia [14,15]. The HOD is a radiological diagnosis based on the finding of a circumscribed hyperintensity of the ION on MRI T2-weighted or fluid attenuated inversion recovery (FLAIR) sequences—an additional olivary hypertrophy is optional [7]. Epidemiological aspects of HOD are still unknown and therapeutic strategies are merely palliative, which is why primary prevention is of particular importance [5]. Moreover, visibility of HOD on MRI has been discussed to be a radiological indicator and surrogate parameter for damage to contralateral cerebellar structures [16].

Furthermore, operative lesions to the cerebellar outflow pathway, consisting of the cerebellar deep nuclei (including dentate, fastigial and interpositus nuclei) and superior cerebellar peduncle (SCP), have been identified to have harmful effects on complex functional cerebrocerebellar connectivity [17,18]. In this area, strategic lesions to the “anatomic cerebellar bottleneck” can disrupt large functional cerebello-thalamo-cortical networks, which expand via the contralateral thalamic mediodorsal nucleus to the cerebral cortex [17,19,20]. In children, brain tumors commonly arise in the posterior fossa in close topographic proximity of these cerebellar structures [16,21]. Frequently, damage to the inferior vermis, fastigial nucleus and the cerebellar outflow pathway results in the troublesome complication of cerebellar mutism syndrome (CMS) and cerebellar cognitive-affective syndrome (CCAS), formerly known as posterior fossa syndrome [22,23,24,25]. After cerebellar tumor resection, about 25 percent of children experience symptoms such as emotional lability, executive dysfunction, language impairment and severely diminished speech or mutism [21,25,26,27]. While the mutism is transient, CMS is widely considered a clinical risk factor for poorer long-term cognitive outcomes, though not without controversy [28,29,30,31,32].

Clearly, the idea that cerebellar injury is negligible is outdated: operative disruption of cerebellar projections to the thalamo-cortical networks can lead to HOD and a wide range of cognitive, memory and affective impairments [33,34]. The fact that the cerebello–thalamo–cortical (also termed dentato–thalamo–cortical) pathway involved in CMS development shares its proximal structures with the GMT (or DROP) responsible for HOD, a connection between the occurrences of the two entities is well conceivable and frequently discussed in the literature [10,35]. However, the risk reduction of operative injury to the efferent cerebellar pathways is undisputable paramount in the prevention of both, CMS and HOD [36,37]. Preventing such surgical injury in posterior fossa surgery is very complex due to the close proximity and exposure of cerebellar structures. It poses a formidable challenge to the neurosurgeon, who needs to be familiar with cerebellar topography and tractography [10,38,39].

In review of the literature and consideration of the cerebellar topography, we hypothesized that a trans-cerebellar and paravermal surgical approach is most likely to injure the DN and consequently affect the GMT [5,6]. The goal of this study is to compare the paravermal trans-cerebellar operative approach to other operative approaches in posterior fossa tumor surgery and to analyze the associated risk of developing HOD on post-operative MRI as well as CMS in the aftermath.

## 2. Results

### 2.1. Patient Characteristics

**Figure 2:**
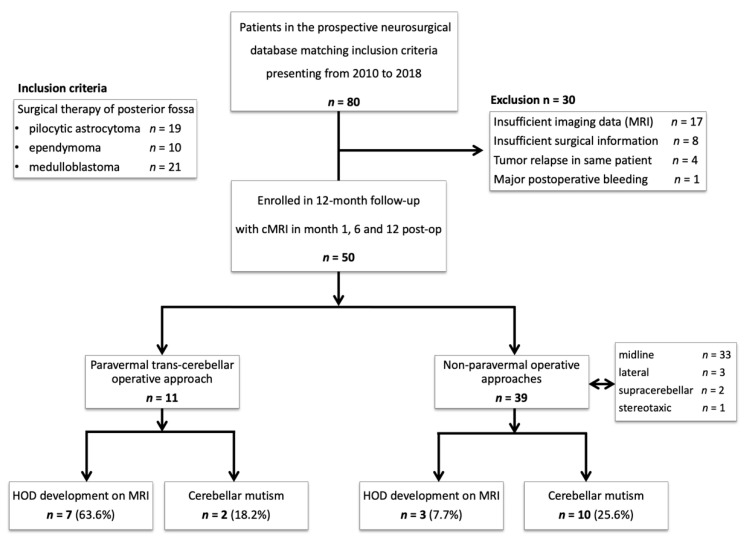
Study protocol: Flowchart diagram showing the enrollment protocol of the study, inclusion and exclusion criteria. Abbreviations: HOD = hypertrophic olivary degeneration; cMRI = cranial MRI.

The identification of our patient cohort as well as the criteria for inclusion and exclusion are shown in Figure 2. Overall, 50 patients were included in our analysis. Neurosurgeons chose a paravermal trans-cerebellar approach in 11 patients. Other approaches comprised of midline approaches in 33 patients (*n* = 23 telovelar, *n* = 10 transvermian), lateral approaches (*n* = 3), supra-cerebellar approaches (*n* = 2) and one stereotaxic. Mean age was 22.7 (±16.9) years, 58.8% (*n* = 30) of patients were male. Histologically, 19 posterior fossa masses were identified as pilocytic astrocytoma WHO grade I, nine as (sub-) ependymoma WHO grade I–III, and 22 as medulloblastomas WHO grade IV. Characteristics of the cohort are outlined in Table 1.

### 2.2. Surgical Approach and HOD Development

**Figure 3:**
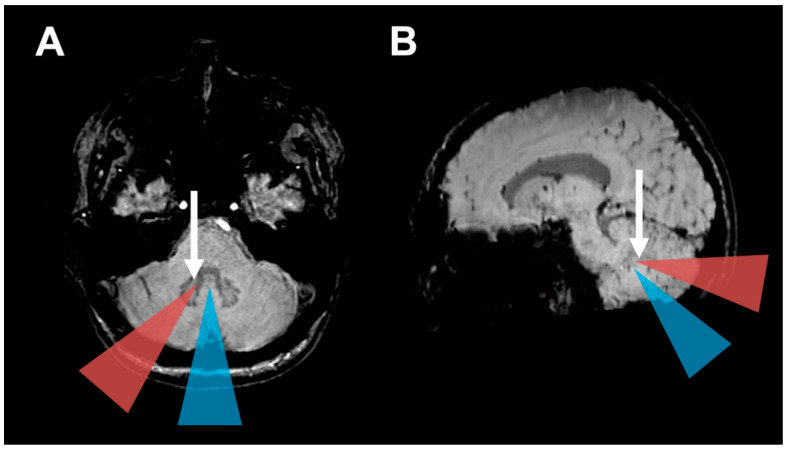
HOD development based on operative approach: (**A**) Axial and (**B**) parasagittal magnetic resonance susceptibility-weighted images (SWI) illustrate the operative approaches investigated in this study. The blue triangles indicate midline approaches (telovelar and transvermian). The orange triangles indicate the paravermal trans-cerebellar operative corridor that frequently led to hypertrophic olivary degeneration in our cohort by injury to the dentate nucleus (Arrows).

Seven out of the eleven patients (63.6%) with a paravermal operative approach developed HOD on MRI, compared to only three of 39 patients (7.7%) with other approaches. This difference in HOD occurrence depending on the operative approach was significant (*p* < 0.001). Consistently, a paravermal approach induced major injury to the DN in 8 out of 11 patients (72.7%), while other approaches mostly spared the DN with an injury occurring only in 13 out of 39 patients (33.3%; *p* = 0.04). Mean tumor volume as assessed on MRI was larger in cases with a paravermal approach (40.8 cm^3^) when compared to other operative approaches (23.6 cm^3^; *p* = 0.05). An associated cyst was more often found in patients with paravermal approach (*p* = 0.02). A posterior fossa tumor localization at the 4th ventricle was found in 33 patients (66%), without a difference between chosen approaches (*p* = 0.73). The association of the paravermal operative approach and HOD development was not confounded by 4th ventricle localization, age, sex, tumor size, chemotherapy or radiotherapy, according to results of the multivariate logistic regression (see Table A1 and Table A2). In head-to-head comparison of midline telovelar and transvermian approaches, no significant differences concerning HOD development (*p* = 1.0, OR 0.86) nor DN injury (*p* = 0.43; OR 0.44) or CMS occurrence (*p* = 1.0; OR 1.0) were found. Figure 3 illustrates the different operative approaches in relation to the dentate nuclei visualized on axial MRI.

### 2.3. HOD-Patient Characteristics and Disease Pattern

Of the 50 patients eligible for analysis, ten patients (20%) developed HOD on subsequent brain-MRI within 12 months post-surgery (Table 2). HOD was located six times on the right, two times on the left and in two cases bilaterally. The presence of HOD was always associated with a distinct contralateral DN lesion (*p* < 0.001, Figure 3). The latency from tumor resection to HOD occurrence on MRI ranged from two to ten months (mean 4.6 months, median 4 months).

**Figure 4:**
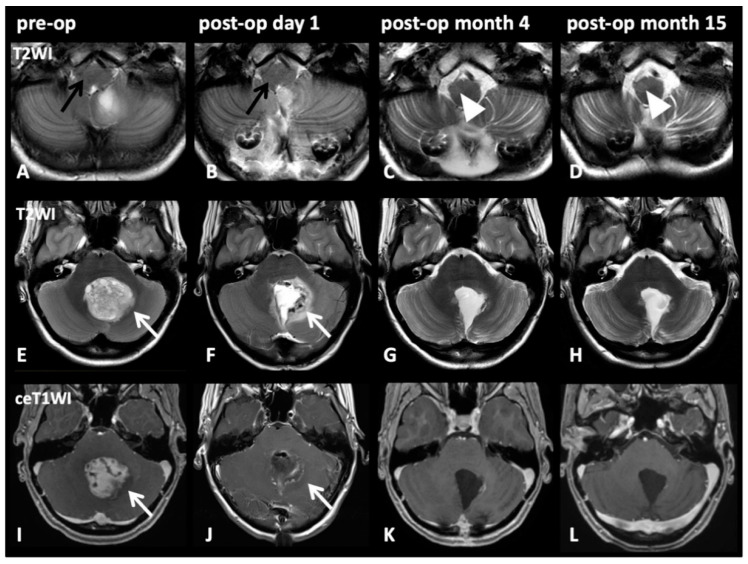
Image series on patient number five from Table 2: Pre-op (1st column), post-op day 1 (2nd column), post-op month 3 (3rd column), post-op month 14 (4th column). T2WI axial slices on medullary level (**A**–**D**) and on pontine level (**E**–**H**) with corresponding pontine axial contrast enhanced T1WI (**I**–**L**). A 17-year-old female patient presented with cerebellar pilocytic astrocytoma affecting the left dentate nucleus (**E**,**I**; white arrows). Initially (**A**; black arrow) and after complete resection via a paravermal approach 6 days later (**F**,**J**; white arrows), no HOD is visible (**B**; black arrow). However, after 3 months (**C**, white arrow) and still after 14 months (**D**; cropped image, arrowheads), HOD with hyperintensity on T2WI on the right side can be found. No signs of tumor recurrence but only discrete scarring is visible after 3 and 14 months (**K**,**L**).

The image series in Figure 4 depicts representative cases of HOD development after tumor resection through a paravermal approach with DN injury. Even though cerebellar pilocytic astrocytoma affected the dentate nucleus preoperatively, no signs of HOD were visible on imaging prior to surgical intervention nor on initial postoperative imaging acquired within one week after intervention. However, radiological signs of HOD were apparent on follow-up MRI within the expected time window three months after the index event and HOD was still visible on follow-up MRI 14 months later.

### 2.4. Surgical Approach and CMS

In 12 patients (24%), the diagnosis of postoperative CMS was made independently based on clinical assessment notes by the treating physicians, synonymously described as “posterior fossa syndrome” [21]. The twelve CMS patients had a median age of 9 years (range 1–16). In patients younger than 18 years, 12 out of 27 (44.4%) developed postoperative CMS. Ten CMS patients (83.3%) were operated via a midline approach (transvermian *n* = 3; telovelar *n* = 7). One patient operated via a paravermal operative approach developed CMS. However, frequency of CMS occurrence was not different between paravermal (18.2%) and other (25.6%) operative approaches (*p* = 1.0). Out of the twelve CMS patients, six showed a major lesion to the DN on postoperative MRI, however, having a DN lesion was not significantly associated with CMS (*p* = 0.74). Only one CMS patient showed signs of HOD on MRI. In two CMS patients, additional postoperative cognitive-affective alterations (CCAS) with attentive deficits were documented. However, not all patients regularly received postoperative testing for specific signs of CCAS and a follow-up neurocognitive testing did not take place in our center.

## 3. Discussion

Our study demonstrates that paravermal trans-cerebellar operative approaches are associated with a significantly higher likelihood of HOD occurrence on MRI than other operative approaches in the context of posterior fossa tumor resection. This can be attributed to operative injury to the DN, which is more frequently inflicted by paravermal approach. This goes in agreement with the literature describing injuries in the GMT as the pathomechanism for the development of HOD [1,7]. In contrast, the midline (telovelar and transvermian), lateral or supra-cerebellar approaches mostly spared the DN, thus seldomly causing HOD. Priorly, case series had indicated that interventional treatment with damage to the GMT is predominantly responsible for HOD occurrence in neuro-oncological patients, and not tumor growth [5,6]. This study supports this concept, as one in five patients developed HOD post-op, but none of these tumor patients showed signs of HOD on MRI prior to neurosurgery. Another finding lies in the lack of a major correlation between HOD and CMS development—a connection that was previously indicated in the literature due to the topographical overlap of the GMT (or DROP) and the cerebello-thalamo-cortical pathways implicated in CMS [10,35,38].

In clinical routine, patients with posterior fossa tumors present with increased intracranial pressure and focal neurological deficits secondary to compression of the brainstem, cranial nerves or cerebellar tissue [41]. Furthermore, due to the tumor anatomy identifying DN is often complicated. Therefore, postoperative outcome parameters comprise of the immediate improvement of these symptoms derived mostly from the reduction of tumor mass effect. In contrast, the symptoms of HOD begin with a delay of months, progress slowly and are easily missed, complicating the allocation to the disease [5]. In concordance with literature, this study found the HOD on MRI with a delay of four months (median) after the contralateral surgical lesion [5,6,7]. Furthermore, most patients are usually not examined clinically in such a depth by neurosurgeons, so that the HOD symptoms may be missed. Some patients with HOD may thus be overlooked in clinical routine and the novel symptoms may mistakenly be attributed to tumor growth or novel pathology [5]. Impedingly, the clinical syndrome and especially palatal tremor is seldom diagnosed. Postoperative speech pathologist exam including fiberoptic endoscopic evaluation of swallowing (FEES) is considered a gold standard for detecting palatal tremor, but regular implementation is not yet universal amongst hospitals [14]. Furthermore, radiologists should also be sensitized for the imaging hallmarks of HOD. For a reliable detection of HOD, axial T2-weighted or FLAIR sequences with a slice thickness of at most 4 mm is recommendable. On standard MR-sequences such as T1-weighted imaging, preoperative identification of the DN can be challenging. However, in our experience the DN can be very well localized on susceptibility weighted imaging (SWI), which we therefore recommend for the neurosurgical planning (see Figure 3). In summary, since the HOD may be associated with a disabling clinical syndrome and therapeutic options are still unavailable, it must be primarily prevented [9,11,13]. Neurosurgeons therefore should consider sparing the DN in the surgical trajectory to prevent HOD occurrence whenever feasible. In some patients another operative trajectory is possible, whereas in other patients a fissure dissecting approach instead of a trans-cerebellar approach should be considered.

In retrospective, different approaches would have been feasible or a different operative strategy should have been chosen for the eleven patients operated via a paravermal trans-cerebellar approach in this study (see Table A3). However, after data analysis of this study we already changed our operative approach concept of posterior fossa tumors in our center. Since mid-2020, we regularly used midline approaches like the telovelar approach or a paramedial supracerebellar approach followed by intracerebellar approach ‘to stay above’ the dentate nucleus for tumor resection and look forward to seeing the long-term results.

Over the past decade, it has become more apparent that the cerebellum is not merely responsible for motor coordination, but that cerebrocerebellar connectivity includes cognitive-associative, affective, executive, linguistic and limbic functions [16,19,42]. In this study, nearly half of patients (44.4%) under 18 years of age developed CMS. This was not associated with injury to the DN nor connected to HOD development. The majority of CMS patients was operated via a transvermian or telovelar approach (83.3%). Arguably, transvermian approaches and cranially extended telovelar approaches caused injury to the inferior vermis, a common cause for CMS [37]. Prior research has indicated that also injury to the DN as part of the cerebellar outflow pathway is a common cause of CMS, which might well explain CMS in the two patients with paravermal approach [17]. However, in this cohort, only half of CMS patients showed DN injury, indicating the presence of multilocular factors in CMS development.

In the literature, presence of bilateral HOD was shown to be strongly correlated with CMS development [10,35]. This finding was consistent with the association between bilateral cerebellar pathway injury and CMS occurrence [36,39]. The small sample size of this study with bilateral HOD does not allow for confirming nor disconfirming this hypothesis: only one patient, a 9-year-old boy, with CMS in this cohort showed additionally signs of a HOD, which was indeed bilateral. The other patient with bilateral HOD, a 34-year-old male, did not show CMS. However, postoperative mutism is rarely seen in adults [21]. In this context it is worth mentioning that also singular lesions alone affecting the crossroads of both GMT in the pons may induce bilateral HOD [12].

Furthermore, the presence of left sided HOD was previously associated with CMS in a combined qualitative and quantitative imaging analysis, which was explained by an injury to the right proximal efferent cerebellar pathway [10]. This functional laterality of CMS was attributed to the disruption of the crossing communication between the right cerebellum and left frontal cortex speech-language processing networks, as earlier proposed by Law and colleagues [20]. In our cohort, the two patients with left sided HOD did not show CMS but were already 32 and 35 years old. Again, as CMS is mostly seen in infants, little conclusion can be drawn from this data.

In summary, a clinically apparent relationship between radiological HOD and CMS development, as hypothesized in the literature, could not be reproduced in our study [10,16,35]. Nonetheless, radiological presence of HOD was always associated with a contralateral postoperative lesion in the DN.

Drawing from these findings, this study indicates that paravermal trans-cerebellar approach frequently equals a “trans-dentate” approach by injuring the DN, while midline, supra-cerebellar and more lateral trans-cerebellar approaches mostly spare it. In terms of DN injury, our study showed that a midline approach is clearly favorable, strengthening the argument made for (fissure dissecting) telovelar and subtonsillar approaches in posterior fossa surgery by Akakin et al. [38]. However, some patients, a telovelar approach may not be feasible due to tumor size, location, and orientation, as demonstrated in this study, where patients with larger tumor volume or an associated cyst significantly more often received a paravermal approach.

Noteworthily, in this study, midline operative approaches led to CMS in 12 out of 27 (44.4%) patients below the age of 18, indicating that vermal injury is a major factor in CMS pathology. The neurosurgical division from the Washington Children’s National Medical Center recently undertook a successful attempt to reduce the incidence of CMS by adopting practices to reducing intraoperative tissue trauma, including minimizing the degree of retraction utilized during the procedure and choosing telovelar approach over transvermian approach to obviate the need for vermial incision [37]. A 2019 international multicenter trial did not reveal surgical factors that predicted postoperative CMS, including surgical hydrocephalus treatment, prone position, ultrasonic aspirator or EVD use, telovelar approach and complete or near total resection [27]. Moreover, a study on 195 pediatric patients using data-driven multivariate lesion symptom mapping besides inferior vermis injury highlighted lesions in the fastigial nuclei, medial DN as well as the SCP as the peak findings in CCAS and CMS patients [17]. Accordingly, sparing the inferior vermis may not suffice to prevent CMS and general intraoperative measures to prevent structural damage should be taken into consideration. In the literature, the reduction of thermal injury (by cavitronic ultrasonic aspirators) and minimization of mechanical injury (by surgical retraction) have been suggested to prevent cerebellar tissue injury [37,43]. Instead, application of slower progressing ‘vascular’ tissue-sparing, fissure dissecting approaches (e.g., telovelar or subtonsilar or supracerebellar) may be favorable [44]. Furthermore, development of neuro-monitoring in analogy to supra-tentorial tumor resection in motor and language systems as well as preoperative fiber tractography imaging were proposed to localize cerebellar structures at risk [16,42].

### Limitations

Limitations of this study include the retrospective design, the limited cohort size as well as missing systematic postoperative neurocognitive testing and inconsistent use of postoperative FEES for definite detection of palatal tremor. Due to retrospective design and duration until occurrence of symptoms, neurosurgeons were not specifically primed to look for HOD-symptoms in postoperative examinations, so that subtle clinical symptoms may have been overlooked. Noteworthily, one patient without later HOD-occurrence was operated by stereotaxy (paramedian trajectory), not by an open corridor approach, and remained in the cohort to comply with study protocol. Furthermore, we had a small amount of missing data for some covariates. Confounder assessment by multivariate logistic regression was therefore only possible with 45 of 50 cases, but we consider it unlikely, that the missing values could profoundly have changed results.

## 4. Materials and Methods

### 4.1. Study Population

We performed a retrospective analysis of our neurosurgical database of patients treated between 2010 and 2018. Inclusion criteria comprised of surgical removal or partial resection of a posterior fossa tumor with either pilocytic astrocytoma, ependymoma or medulloblastoma as subsequent diagnosis. We assessed age, gender, tumor size and localization, histology and treatment as possible prognostic factors. Furthermore, all operations were carefully reviewed (neurosurgical database, medical records and video database) by two experienced neurosurgeons (J.K. and P.B.) to evaluate approach and intra-OP situations. Ethical approval for the study was granted by the institutional Review Board of the Ethical Committee at the University Hospital Frankfurt (project-number: 274/18: UCT-53-2020). Written informed consent of the patients was obtained.

### 4.2. Magnetic Resonance Imaging

Preoperative MRI was available in all patients. Postoperative MRI examinations were conducted within one, six and twelve months after surgery. Examinations during all three time points were available in 43 patients, at least two examinations were available in 5 patients and at least one examination was available in 2 patients. All examinations were reviewed by experienced radiologists (M.W., E.H., E.S.), who were blinded to the clinical outcomes. The radiological diagnosis of HOD was established in consensus if (1) postoperatively a new-appearing, circumscribed hyperintensity of the inferior olivary nucleus was present on T2-weighted or FLAIR (fluid-attenuated inversion recovery) imaging, (2) that was possibly accompanied by hypertrophy of the inferior olivary nucleus and (3) showed no contrast enhancement or diffusion restriction [7,45,46]. The clinical symptoms were independently evaluated by an experienced neurologist (M.S.-P.). In addition, postoperative patterns of cerebellar injury were analyzed. Because MRI scans were acquired for clinical indications, MRI-sequences and time since operation were not homogeneous throughout the cohort.

### 4.3. Statistics

For statistical analysis, the sample was divided into two subsamples based on operative approach either by paravermal trans-cerebellar or other operative approaches. We calculated descriptive statistics on the total sample and the two subsamples. As a measure of association between the operative approach and covariates unadjusted odds ratios (OR) were calculated (for binary variables). *p*-values were calculated by Welch’s two-sample *t*-test (for continuous variables) and by Fisher’s exact test (for binary variables). To exclude potential confounding a multivariate logistic regression predicting HOD development was calculated, which included the operative approach and the most relevant potential confounders as covariates. R in version 3.6.1 (R Foundation for Statistical Computing, 2019, Vienna, Austria) was used for statistical calculations.

## 5. Conclusions

Paravermal trans-cerebellar approach frequently causes HOD on MRI due to injury to the DN when compared to other more midline or lateral approaches, which normally spare the DN. This study further underlines the need for a heightened awareness towards cerebellar functional anatomy, minimization of intraoperative tissue injury and the necessity of thorough and individualized preoperative planning in posterior fossa surgery. While the HOD itself is a potentially disabling disease, it bears further value as a reliable indicator for functional disruption of cerebellar outflow pathways and the DN in particular, with or without resulting CMS. The ideal way to operate cerebellar tumors remains to be determined and further studies are urgently needed. However, we clearly identify that a trans-cerebellar approach more often leads to HOD, so that this approach, if possible, should be avoided.

## Figures and Tables

**Table 1:** Patient matrix. The table shows a patient matrix of paravermal trans-cerebellar approach vs. other operative approaches with absolute numbers and percentages within the given vertical column provided in brackets for clarity. Other operative approaches (*) comprise of midline (*n* = 33), supra-cerebellar (*n* = 2), lateral approach (*n* = 3) and stereotaxy (*n* = 1). Odds ratio is provided where applicable. ^1^ Tumor size volumes were calculated using the ABC/2 estimation formula based on MRI-data [40]. ^2^ Two patients excluded due to missing information. ^3^ The other operational positionings include Fukushima, oblique body position, lateral position. ^4^ Defined as radiological evidence of HOD on MRI; Abbreviations: HOD = hypertrophic olivary degeneration; CMS = cerebellar mutism syndrome; n/a = not applicable.

*n* = 50	All Patients *n* = 50	Paravermal Trans-Cerebellar Approach *n* = 11	Other Operative Approaches * n = 39	*p*-Value	Odds Ratio
Age (mean ± SD)	22.7 ± 16.9	20.1 ± 17.5	23.5 ± 17.0	0.57	
Male sex (*n*)	30	6 (54.5%)	24 (61.5%)	0.74	0.75
Tumor size ^1^ (cm^3^ ± SD)	27.6 ± 20.6	40.8 ± 26.4	23.6 ± 16.9	0.05	
Histology:					
- astrocytoma	19	7 (63.6%)	12 (30.7%)	0.08	3.94
- ependymoma	9	1 (9.1%)	8 (20.5%)	0.66	0.39
- medulloblastoma	21	3 (27.2%)	18 (46.1%)	0.32	0.44
Chemotherapy	24	4 (36.3%)	20 (51.2%)	0.50	0.54
Radiotherapy ^2^	27	4 (36.3%)	23 (58.9%)	0.17	0.35
Associated cyst	13	6 (54.5%)	7 (17.9%)	0.02	5.49
Location 4th ventricle	33	8 (72.7%)	25 (64.1%)	0.73	1.49
Operative positioning:					
- Semi-sitting	30	6 (54.5%)	24 (61.5%)	0.09	0.29
- Prone	15	4 (36.3%)	11 (28.2%)	0.71	1.45
- other ^3^	4	3 (27.2%)	1 (2.6%)	0.03	14.25
Dentate nucleus injury	21	8 (72.7%)	13 (33.3%)	0.04	5.33
HOD development ^4^	10	7 (63.6%)	3 (7.7%)	0.0003	21
Cerebellar mutism					
- all	12	2 (18.2%)	10 (25.6%)	1.0	0.64
- <18 years	12	2 (18.2%)	10 (25.6%)	0.41	0.40

**Table 2:** HOD patient baseline data. The characteristics of ten patients with HOD development on MRI are shown. Age is defined as age at the time of the initial diagnosis of the brain tumor. Diagnosis is based on histopathology. The surgical approaches are listed as taken from the OR-reports. Latency resection to HOD is defined as time in between the first surgical operation to first radiological signs of HOD on MRI, months are rounded up for >2 weeks. HOD symptoms are defined as palatal tremor, Holmes-tremor and pendular nystagmus. Tumor size volumes were calculated using the ABC/2 estimation formula based on MRI-data [40]. Abbreviations: Pat. = Patient; mo. = months; CMS = cerebellar mutism syndrome; m = male; f = female; SCP = superior cerebellar peduncle.

Pat.	Age, Sex	Diagnosis WHO°	HOD Side	Tumor Localization	Operative Approach	Lesion within GMT	Adjuvant RCT	Tumor Size (mm) [Volume]	Time Lesion to HOD	CMS
1	20, f	pilocytic astrocytoma I°	right	4th ventricle	paravermal trans-cerebellar	both dentate nuclei (left > right)	none	63 × 42 × 36 [48 cm^3^]	2 mo.	no
2	60, f	pilocytic astrocytoma I°	right	4th ventricle to vermis	paravermal trans-cerebellar	both dentate nuclei (left > right)	none	42 × 63 × 56 [74 cm^3^]	8 mo.	no
3	32, m	pilocytic astrocytoma I°	left	4th ventricle to vermis	paravermal trans-cerebellar	right dentate nucleus	none	37 × 40 × 30 [22 cm^3^]	6 mo.	no
4	14, f	pilocytic astrocytoma I°	right	cerebellar vermis	paravermal trans-cerebellar	both dentate nuclei	none	58 × 57 × 57 [94 cm^3^]	4 mo.	no
5	17, f	pilocytic astrocytoma I°	right	4th ventricle	paravermal trans-cerebellar	left dentate nucleus	none	33 × 34 × 39 [22 cm^3^]	4 mo.	no
6	35, m	ependymoma II°	left	4th ventricle	midline telovelar	both dentate nuclei (right > left)	none	42 × 15 × 20 [6 cm^3^]	3 mo.	no
7	14, m	medulloblastoma IV°	right	4th ventricle	midline transvermian	left dentate nucleus	yes	34 × 47 × 37 [30 cm^3^]	3 mo.	no
8	34, m	medulloblastoma IV°	bilateral	4th ventricle	midline telovelar	both dentate nuclei	yes	46 × 35 × 35 [28 cm^3^]	10 mo.	no
9	9, m	medulloblastoma IV°	bilateral	4th ventricle and cerebellum	paravermal trans-cerebellar	both dentate nuclei, left SCP	yes	53 × 37 × 48 [47 cm^3^]	4 mo.	yes
10	40, m	medulloblastoma IV°	right	4th ventricle	paravermal trans-cerebellar and telovelar	left dentate nucleus	yes	31 × 21 × 27 [9 cm^3^]	3 mo.	no

## Data Availability

The datasets analyzed during the current study are available from the corresponding author upon reasonable request.

## Appendix A

**Table A1:** Multivariate logistic regression. The multivariate logistic regression predicting HOD development is shown, due to partially missing information the analysis was run on *n* = 45.

Variable	Odds Ratio	*p*-Value
Paravermal trans-cerebellar approach	39.26	0.0041
Age	1.06	0.1059
Sex	1.90	0.5646
Volume	1.00	0.4982
Chemotherapy	0.42	0.5743
Radiotherapy	4.83	0.3133

**Table A2:** Multivariate logistic regression predicting HOD development (*n* = 45) based on 4th Ventricle localization. The multivariate logistic regression predicting HOD development is shown, due to partially missing information the analysis was run on *n* = 45.

Variable	Odds Ratio	*p*-Value
Paravermal trans-cerebellar approach	54.72	0.008
Age	1.07	0.110
Sex	1.89	0.588
Volume	1.00	0.285
Chemotherapy	0.84	0.919
Radiotherapy	5.79	0.298
Location 4th Ventricle	5.09	0.294

**Table A3:** New recommended approaches in retrospective analysis of the preoperative situations of all eleven patients with a paravermal trans-cerebellar approach in this study. Later HOD refers to the occurrence of HOD on cMRI within 12 month-follow up. Abbreviations: DN = dentate nucleus.

Age, Sex	Diagnosis WHO°	Later HOD	Tumor Localization	Associated Cyst	Original Operative Approach	New Recommended Approach
12, m	pilocytic astrocytoma I°	no	4th ventricle to cerebellum	yes	paravermal trans-cerebellar	telovelar
20, f	pilocytic astrocytoma I°	yes	4th ventricle	no	paravermal trans-cerebellar	telovelar
12, f	pilocytic astrocytoma I°	no	4th ventricle	yes	paravermal trans-cerebellar	supracerebellar, then transcerebellar
2, m	ependymoma II°	no	4th ventricle	no	paravermal trans-cerebellar	combined telovelar and retrosigmoid
60, f	pilocytic astrocytoma I°	yes	4th ventricle to vermis	yes	paravermal trans-cerebellar	original approach, but without cyst membrane removal
32, m	pilocytic astrocytoma I°	yes	4th ventricle to vermis	yes	paravermal trans-cerebellar	telovelar
14, f	pilocytic astrocytoma I°	yes	vermis	yes	paravermal trans-cerebellar	supracerebellar, then transcerebellar
17, f	pilocytic astrocytoma I°	yes	4th ventricle	no	paravermal trans-cerebellar	telovelar
3, m	medulloblastoma IV°	no	cerebellum	yes	paravermal trans-cerebellar	original approach, but without cyst membrane removal
9, m	medulloblastoma IV°	yes	vermis and cerebellum	no	paravermal trans-cerebellar	original approach, respect DN as feasible
40, m	medulloblastoma IV°	yes	4th ventricle	no	paravermal trans-cerebellar	telovelar
